# Development and translation of biodegradable metal stents: from heart to brain

**DOI:** 10.1093/rb/rbag079

**Published:** 2026-05-29

**Authors:** Xiaofeng Cao, Guanjun Chen, Yuchen Fan, Yunong Shen, Ming Li, Yufeng Zheng

**Affiliations:** School of Materials Science and Engineering, Peking University, Beijing 100871, China; School of Materials Science and Engineering, Peking University, Beijing 100871, China; School of Materials Science and Engineering, Peking University, Beijing 100871, China; School of Materials Science and Engineering, Peking University, Beijing 100871, China; Neuro-Cardiovascular Research Center, Xuanwu Hospital, Capital Medical University, Beijing 100053, China; School of Materials Science and Engineering, Peking University, Beijing 100871, China

**Keywords:** biodegradable metals, zinc alloy stents, magnesium alloy stents, iron alloy stents, cerebrovascular diseases

## Abstract

Biodegradable metal stents offer a promising approach for vascular intervention by providing temporary mechanical support and subsequently degrading once healing is complete. While significant progress has been made in cardiovascular applications, their adaptation for cerebrovascular use remains an active area of research. This review systematically examines three key biodegradable metal systems: Fe, Zn and Mg alloys. It traces their development and specific considerations in the transition from cardiac to cerebral indications. Fe-based stents exhibit high mechanical strength but degrade slowly, prompting strategies to accelerate corrosion. Zn-based stents provide a more moderate degradation rate and favorable biocompatibility, yet their neurovascular safety profile requires further assessment. Mg-based stents, which degrade rapidly and may confer neuroprotective benefits, have advanced into clinical use, though controlling their degradation kinetics remains essential. Translating these stents to the delicate and tortuous cerebrovascular environment introduces specific challenges, including the need for enhanced anatomical conformability, mitigation of potential neurotoxicity from degradation byproducts, and reduction of MRI artifacts. Future development will depend on material-specific strategies: accelerating degradation for Fe alloys, refining controlled-release coatings for Zn alloys, and leveraging the neuroprotective potential of Mg alloys. Ultimately, success hinges on optimizing stent degradation and neurovascular compatibility.

## Graphical abstract

**Graphical Abstract:**
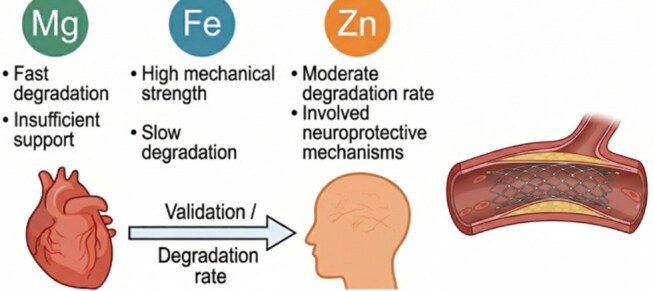

## Degradable metal stents in vascular medicine

**Figure 1:**
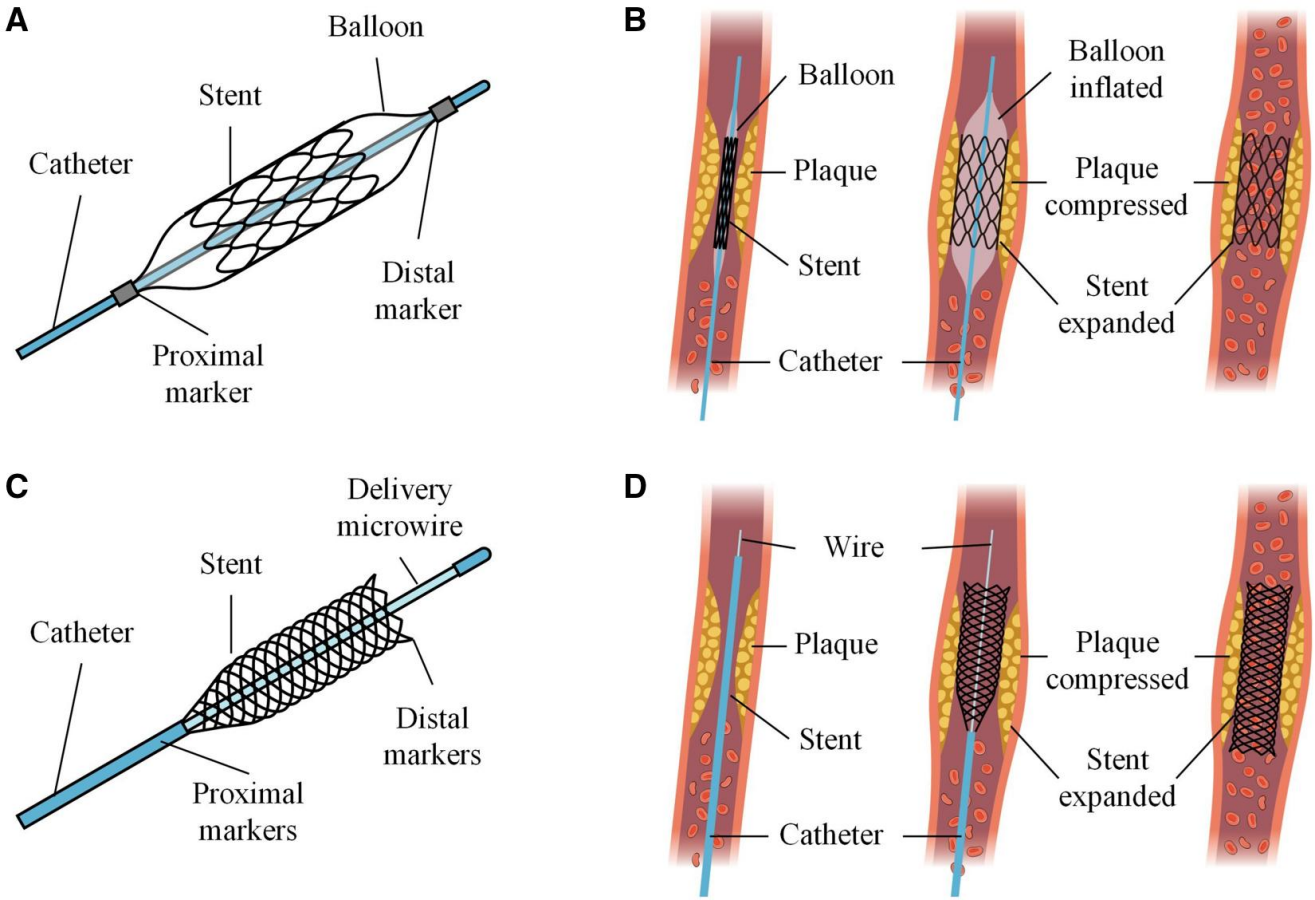
Deployment methods of vascular stents. (**A**) Schematic of the balloon-expandable stent. (**B**) Deployment of the balloon-expandable stent. Balloon inflation expands the stent to support the vessel and balloon deflation leaves the stent in place. (**C**) Schematic of the self-expanding stent. (**D**) Deployment of the self-expanding stent. As the sheath gradually retracts, the stent expands and apposes to the vessel wall.

Interventional stent implantation has become a standard therapeutic strategy for vascular conditions such as coronary and peripheral artery stenosis. Vascular stents can provide sustained mechanical support to the vessel wall, thus reducing the risk of acute vessel occlusion and facilitating revascularization. These stents are commonly made of metallic materials, such as stainless steel, cobalt-chromium alloys [1] and nickel-titanium alloys due to their excellent mechanical properties, biocompatibility and suitability for precise fabrication [2]. Clinically, vascular stents are implanted via balloon expansion [3] or self-expansion, tailored to the specific vascular environment, as shown in Figure 1. Although the stent materials are biologically inert, they may still induce irritation to the surrounding tissues after implantation, potentially inhibiting endothelial cell growth and leading to complications such as delayed thrombosis, long-term restenosis or atherogenesis. In addition, the corrosion and abrasion of stents can lead to the release of metal ions, which may trigger allergic and inflammatory reactions in the body [4].

Drug-eluting stents (DES) were developed to alleviate in-stent restenosis after bare-metal stent (BMS) implantation. By applying a drug-releasing layer onto the metallic scaffold, DES can suppress the excessive proliferation of vascular smooth muscle cells, thereby reducing the incidence of restenosis. However, first-generation DES were associated with an increased risk of late stent thrombosis (LST) due to delayed arterial healing and poor re-endothelialization [5]. Second-generation DES addressed the issue with thinner struts, optimized polymer coatings, and novel antiproliferative agents [6]. Moreover, third-generation DES have introduced biodegradable polymer coatings or polymer free designs to further minimize the risk of LST and inflammation [7]. Despite these advancements, the permanent metallic backbone remains a foreign body within the vessel, which may still trigger chronic inflammation, impair complete endothelialization, and limit the feasibility of future reintervention.

To overcome the limitations associated with permanent stent implantation, biodegradable stents are being explored by providing temporary mechanical support and gradually degrading after vessel healing is complete. Polymer-based biodegradable scaffolds, exemplified by poly-L-lactic acid (PLLA)-based devices such as the ABSORB BVS (Abbott) [8], showed early clinical promise but were limited by poor mechanical properties and unpredictable rate of degradation. Larger randomized controlled trials showed higher risk of stent thrombosis, failing to demonstrate clear clinical benefit of polymer-based scaffolds over metallic DES [9]. Increasing attention has been directed toward biodegradable metallic stents, which offer superior mechanical properties, more controlled degradation behavior and better biocompatibility.

**Figure 2:**
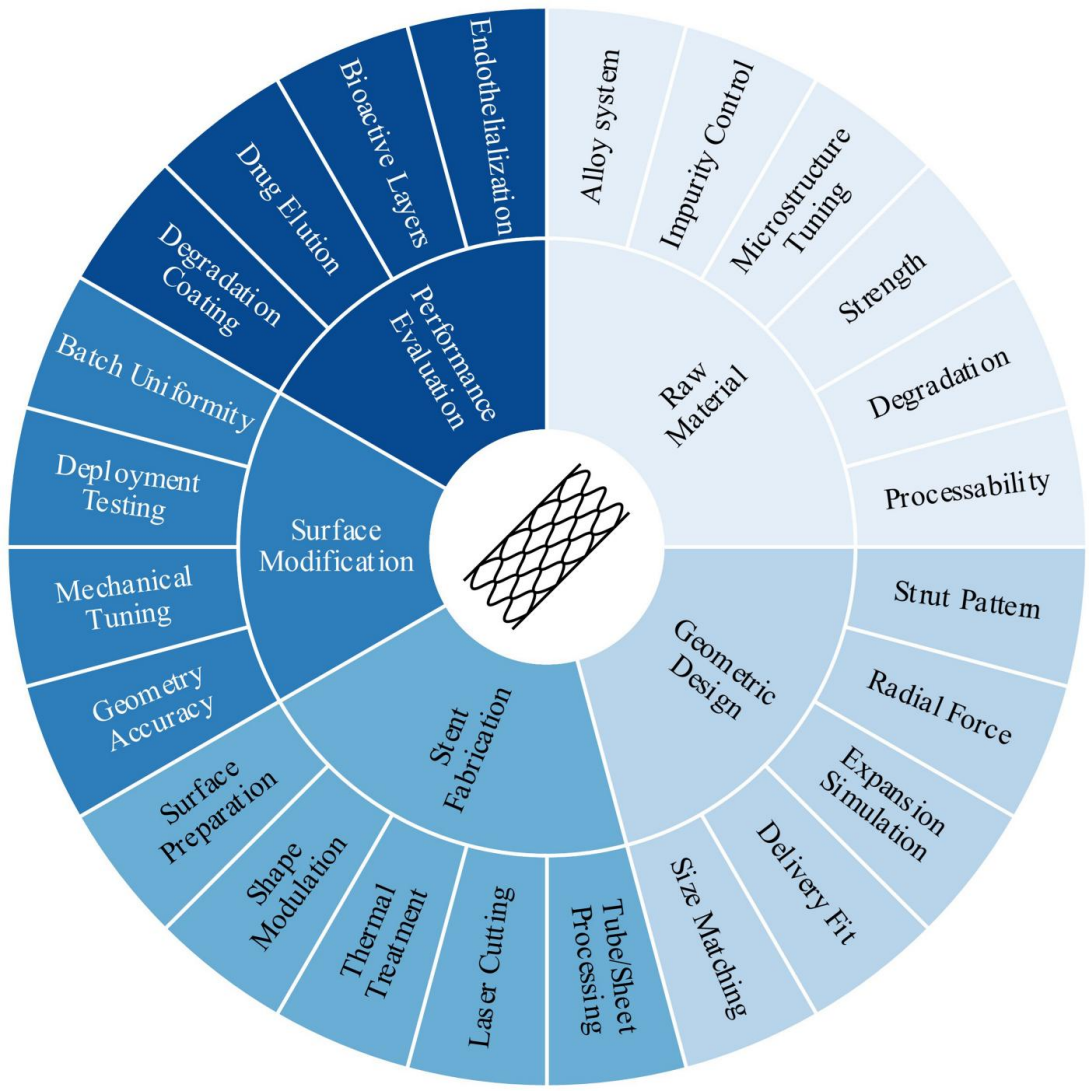
Biodegradable metal stent design considerations.

The metals most frequently employed for biodegradable stents are magnesium (Mg)-, iron (Fe)-, or zinc (Zn)-based alloys. The processing of these materials for use as degradable metal stents is optimized to possess sufficient mechanical support, an appropriate degradation rate, and good biocompatibility (Figure 2) [10]. A critical step in the fabrication of stents is the processing of thin-walled capillary tubes. This process can be challenging, especially for Mg and Zn, even though their ductility is generally better than that of current biostable stent materials [11]. To optimize the stent structure to match the requirements of the blood vessel, finite element analysis is used to model the stress distribution and degradation behavior of the stent in the vascular environment. The stents are then prepared via machining and laser cutting, followed by surface polishing and sterilization.

**Figure 3:**
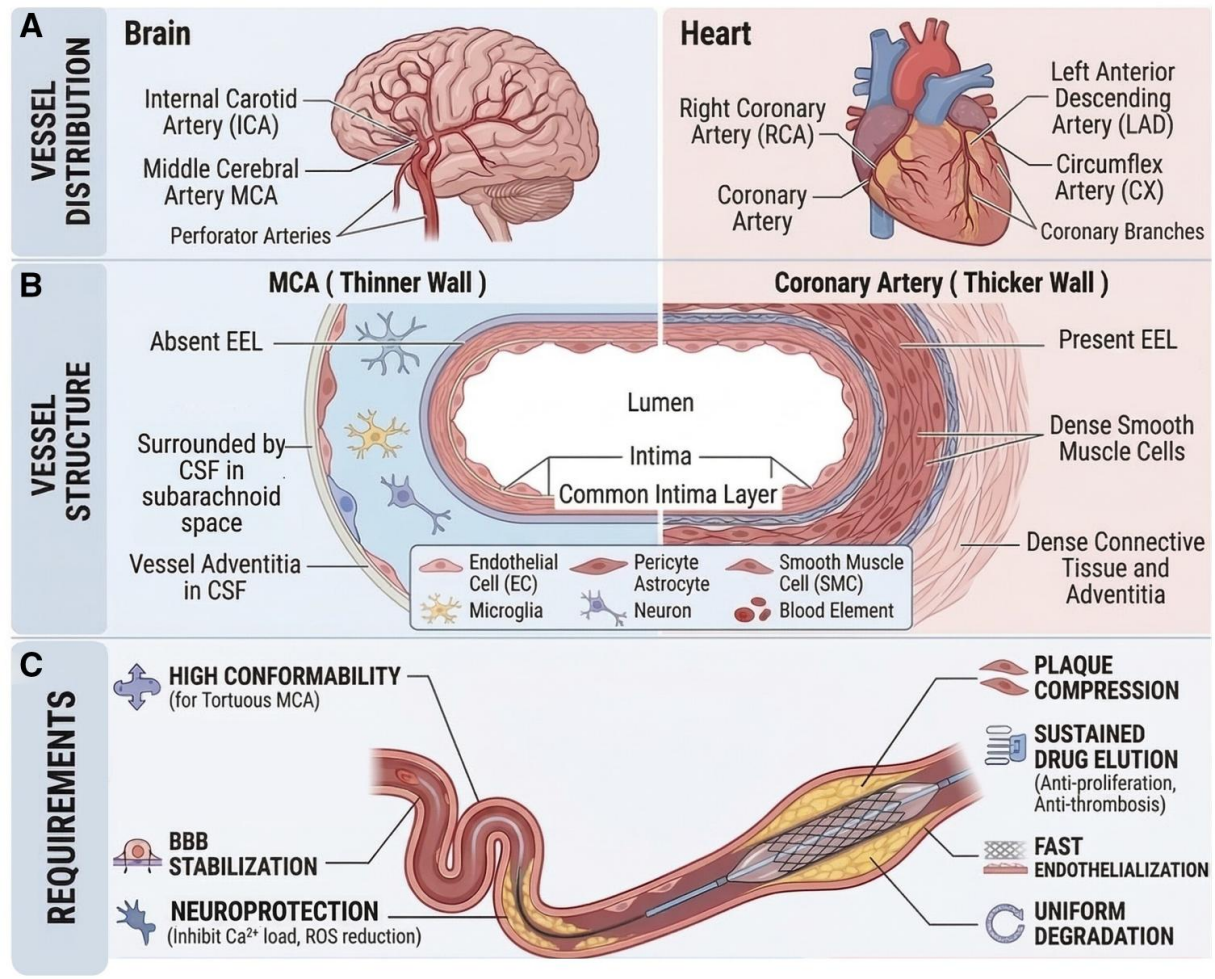
Comparative analysis of intracranial and coronary vascular environments and implications for specialized stent design. (**A**) Schematic of vessel distribution in the brain and heart. (**B**) Cross-sectional structure comparison, highlighting the relatively thinner wall, absence of an EEL, and unique neurovascular unit in the MCA versus the thicker-walled coronary artery. (**C**) Distinct stent requirements, contrasting the high conformability and proposed neuroprotection needed for intracranial applications with the plaque compression and drug elution required for coronary stents.

Beyond the specific stent structural designs discussed in Figure 2, the successful clinical application of biodegradable cerebrovascular stents is fundamentally predicated on addressing the stark contrasts between the intracranial and coronary physiological environments (Figure 3). As systematically illustrated, intracranial vessels like the middle cerebral artery possess a distinctly fragile structure, characterized by a relatively thinner wall and the absence of an external elastic lamina (EEL), and are suspended within the cerebrospinal fluid of the subarachnoid space, rather than being embedded in dense tissue. These unique anatomical and localized physiological constraints profoundly dictate that cerebrovascular stents must fulfill entirely distinct mechanical and biological performance criteria, prioritizing factors such as extreme conformability to navigate severe tortuosity and strict localized neuroprotection.

Multiple designs of biodegradable metal stents have been developed for cardiovascular applications and several are now being commercialized. Among these, degradable Mg-based alloy stents have undergone extensive animal experimentation and clinical testing, demonstrating therapeutic efficacy and clinical safety [12]. The Magmaris^®^ degradable Mg alloy stent, produced by Biotronik, obtained the world’s first CE (Conformité Européenne) certification in 2016 [13]. Degradable Fe-based alloy stents have also progressed into the clinical trial phase, with the Fe-based IBS^®^ absorbable drug-eluting coronary stent system developed by Lifetech Scientific obtaining approval in China in 2021 [14]. In recent years, Zn-based alloys have emerged as a promising class of biodegradable metals due to its excellent biocompatibility and degradation rate. Although biodegradable Zn-based alloy heart stents are still in the preclinical stage, current efforts are laying the foundation for future clinical trials [11].

Intracranial Atherosclerotic Stenosis (ICAS) is one of the most important causes of ischemic stroke, with a notably high prevalence of 33–50% in Chinese patients [15, 16]. Currently, the main treatment for ICAS is aggressive medical management, including antiplatelet therapy, blood pressure control, and lipid-lowering interventions. Stents have also been considered as a therapeutic option for ICAS. However, unlike in coronary artery disease, intracranial stenting has not demonstrated clear superiority in large-scale clinical trials [17] and is currently reserved for high-risk patients who fail to respond adequately to medical therapy. Moreover, permanent metal stents may cause a chronic inflammatory response and damage the elasticity of the vessel wall, leading to increased luminal stenosis, interfering with normal vascular remodeling and potentially affecting future therapeutic options.

## Biodegradable Fe-based alloy stents

### Fe in the human body and Fe-based alloys

Systemic toxicity by Fe-based stents is relatively low due to their excellent biocompatibility, slow degradation rate and their low mass (approximately 40 mg) [18]. Fe is an essential trace element whose estimated required daily intake for adults is about 1 mg. The human body contains about 5 g of iron, approximately 75% of which is found within hemoglobin, a key carrier of O_2_ [19]. Fe also serves as a cofactor for many enzymatic reactions, including DNA synthesis and oxidoreductase. In addition, the mechanical properties of degradable Fe-based stents are outstanding. Fe-based alloys have a high modulus of elasticity and strong radial support, permitting the use of very thin walls (∼53 μm) in their stents [20] to maintain the opening of the blood vessel while minimizing disruption to intravascular blood flow.

The degradation of Fe-alloy stents in blood vessels primarily occurs through electrochemical corrosion, local acidic environments, and mechanical stress, leading to the release of Fe^2+^ and Fe³^+^ ions, which further form oxides such as Fe_2_O_3_ and Fe_3_O_4_. These oxides gradually degrade through chemical dissolution, biodegradation and mechanical wear. Meanwhile, the vascular healing process proceeds in parallel, involving plasma protein adsorption, endothelial cell adhesion, proliferation, and migration, which ultimately lead to the formation of a continuous neointima that restores vascular function. This favorable healing response underlines the good biocompatibility of Fe-alloy stents. The inflammatory response induced during degradation is generally controllable, and successful endothelialization further reduces the risks of thrombosis and restenosis, ensuring the safety and reliability of Fe-alloy stents in clinical applications.

### Clinical studies of degradable Fe-based stents

Fe was the first metallic material used for degradable stents; however, it typically requires over two years to completely degrade. To accelerate their degradation, new Fe alloys are being developed with more optimal corrosion profiles. Early implantation studies of Fe stents in animal models revealed the implants can induce mild-to-moderate inflammatory responses with variable individual reactions, but there has yet to be evidence of Fe toxicity [21]. The development of iron-based biodegradable stents began with the pioneering study by Peuster et al. [22] in 2001, who first implanted high-purity iron (>99.8%) stents into rabbit aortas, preliminarily verifying their *in vivo* safety. Subsequent research has primarily focused on animal studies, such as implantation in porcine coronary arteries [23]. To further improve the degradation performance and biocompatibility of iron, researchers developed surface modification strategies, such as using polylactic acid (PLA) coating to accelerate the corrosion of the iron substrate through its acidic degradation products, while enabling local drug delivery [24]. In recent years, novel iron-based stents such as the PDLLA-Zn-nitrided iron stent have demonstrated complete degradation within 24 months in both rabbit aortas and human coronary arteries, showing controllable degradation and good biocompatibility [25].

However, despite the potential of iron-based stents in terms of mechanical properties and controllable degradation, their biocompatibility—particularly their potential toxicity to nerve cells—has not been fully elucidated, limiting their application in neurovascular fields [26]. In December 2019, the IBS^®^ Absorbable Drug-Eluting Coronary Stent System developed by Yuanxin Technology (Shenzhen) Co., Ltd., a subsidiary of Xianjian Technology, successfully completed its First-in-Human (FIM) clinical enrollment in Fu Wai Hospital of the Chinese Academy of Medical Sciences. In the study, the team implanted IBS stents in 45 patients, all of whom had a 100% success rate with no intraoperative complications. The product consists of nitrided pure iron (Fe-0.05% N) as its backbone, with zinc and polymer coatings to accelerate the degradation rate of the iron, as well as the drug sirolimus. The total wall thickness of the stent struts is approximately 70 μm, which is comparable to the diameter of a human hair (Figure 3) [27].

### Considerations for intracranial application of Fe-based stents

Iron plays a critical role in the nervous system, supporting neurotransmitter synthesis, myelination and mitochondrial function. It is particularly significant in energy production and cell signaling processes. However, dysregulation of iron in the brain can lead to its accumulation, and in turn, trigger oxidative stress and inflammation, manifestations that are closely associated with neurodegenerative diseases including Alzheimer’s [28]. The blood–brain barrier (BBB) tightly regulates brain Fe levels through transport proteins. Still, damage to the BBB can result in abnormal iron accumulation, especially when transferrin is dysregulated or systemic iron levels are high, leading to neuroinflammation and cellular damage [29, 30]. Additionally, iron can induce cell death. Ferroptosis is closely associated with lipid peroxidation, particularly when glutathione (GSH) and glutathione peroxidase 4 (GPX4) are compromised [31, 32]. In the brain, where polyunsaturated fatty acids are abundant and prone to oxidation, ferroptosis can lead to extensive neuronal damage [33].

With regard to Fe-based intracranial stent design, iron’s high mechanical strength and elasticity provide effective radial support under vascular pressure [34]. Still, the unique anatomy of intracranial vessels, which are narrow and curved, requires stents that also provide both flexibility and adaptability. Standard coronary stent designs, such as those produced by laser-cutting, may be too rigid, necessitating customized designs to ensure unobstructed blood flow and compatibility with vessel walls [35]. In addition, the magnetic properties of iron cause artifacts in magnetic resonance imaging (MRI), distorting images around the stent site, which impacts the accuracy of diagnostic imaging and monitoring of stented vessels [36]. In clinical and preclinical imaging evaluations, Use MRI directly. compatibility is a crucial determinant for intracranial stent applications. Fe-based stents, due to their innate ferromagnetic properties, generate significant susceptibility artifacts that obscure the surrounding vasculature and brain parenchyma, posing severe challenges for non-invasive postoperative monitoring [10]. Conversely, Mg-based stents exhibit excellent MRI compatibility with minimal artifact generation, allowing for clear, artifact-free visualization of the stented vessel and adjacent neural tissues, thereby offering a distinct advantage for long-term clinical follow-up [37]. Finally, when applied in intracranial vessels, the rate of degradation is too slow and may persist for several years, thus affecting subsequent interventions. Long-term residual metal may trigger a local inflammatory response and increase the risk of vessel restenosis. Therefore, despite the advantages of Fe alloys in the field of large vessels, their application in intracranial vascular stents still faces many challenges.

## Biodegradable Zn-based alloy stents

### Zn in the human body and Zn-based alloys

Zn has emerged as a promising material in the field of degradable metals in recent years. Its degradation rate *in vivo* is between that of Mg and Fe, which is more optimal for biodegradable metal stents [38]. Due to suitable degradation rate, appropriate mechanical properties and excellent biocompatibility, Zn-based materials have emerged as promising candidates for biodegradable stents [39].

Zinc plays a vital role in maintaining physiological functions. Zn accounts for about 0.003% of the body weight in humans, with 90% found in muscles and bones and 10% found in the blood [40]. Zn serves as an activator for multiple enzymes and enhances immunity by promoting phagocytosis and immunoglobulin production. It is also a component of over 300 enzymes involved in numerous physiological processes. In the cardiovascular system, Zn protects cardiomyocytes from acute oxidative stress, prevents inflammatory responses during myocardial injury, promotes wound healing, and enhances the survival of myocardial stem cells during recovery [41, 42]. It also regulates apoptosis and inflammatory responses, decreases the expression of endothelial adhesion molecules, and lowers the risk of atherosclerosis. Zn can also regulate vascular tone and promote vasodilation [43].

The degradation of Zn-alloy stents in blood vessels primarily occurs through electrochemical corrosion, local acidic environments, and mechanical stress, leading to the release of Zn^2+^ ions and the formation of oxides such as ZnO or Zn (OH)_2_. These oxides gradually degrade through chemical dissolution, biodegradation and mechanical wear. The endothelialization process involves plasma protein adsorption, endothelial cell adhesion, proliferation and migration, ultimately forming a continuous neointima that restores vascular function.

### Advancement and frontiers of degradable Zn-based stent development

**Figure 4:**
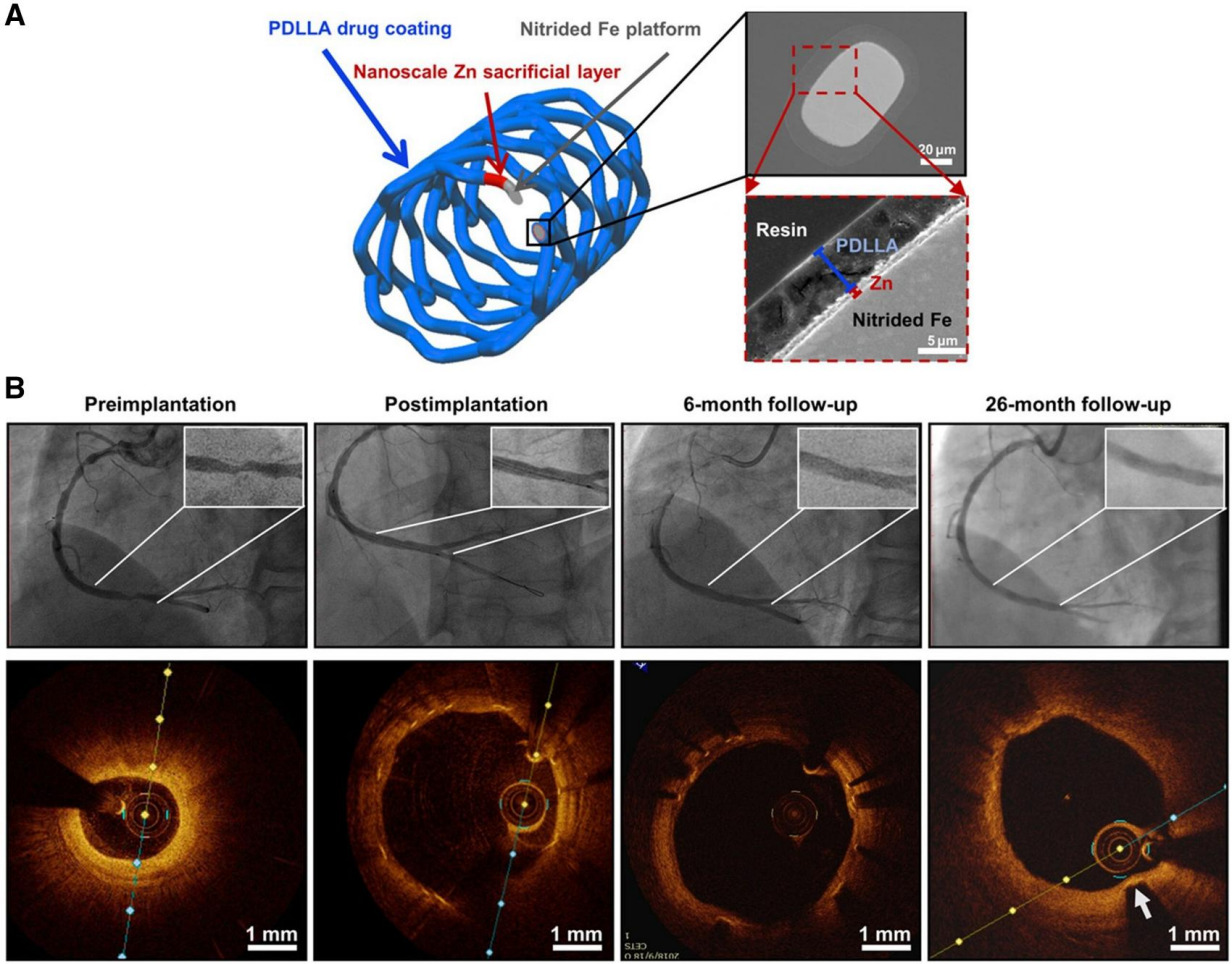
Design and the first in-human implantation of the IBS^®^ absorbable drug-eluting coronary stent. (**A**) The morphology and microstructure characterization of the stent [25]. (**B**) Angiographic images(top) and OCT images(bottom) of the vessel condition at pre-implantation, post-implantation, 6-month and 26-month follow-ups [25].

**Figure 5:**
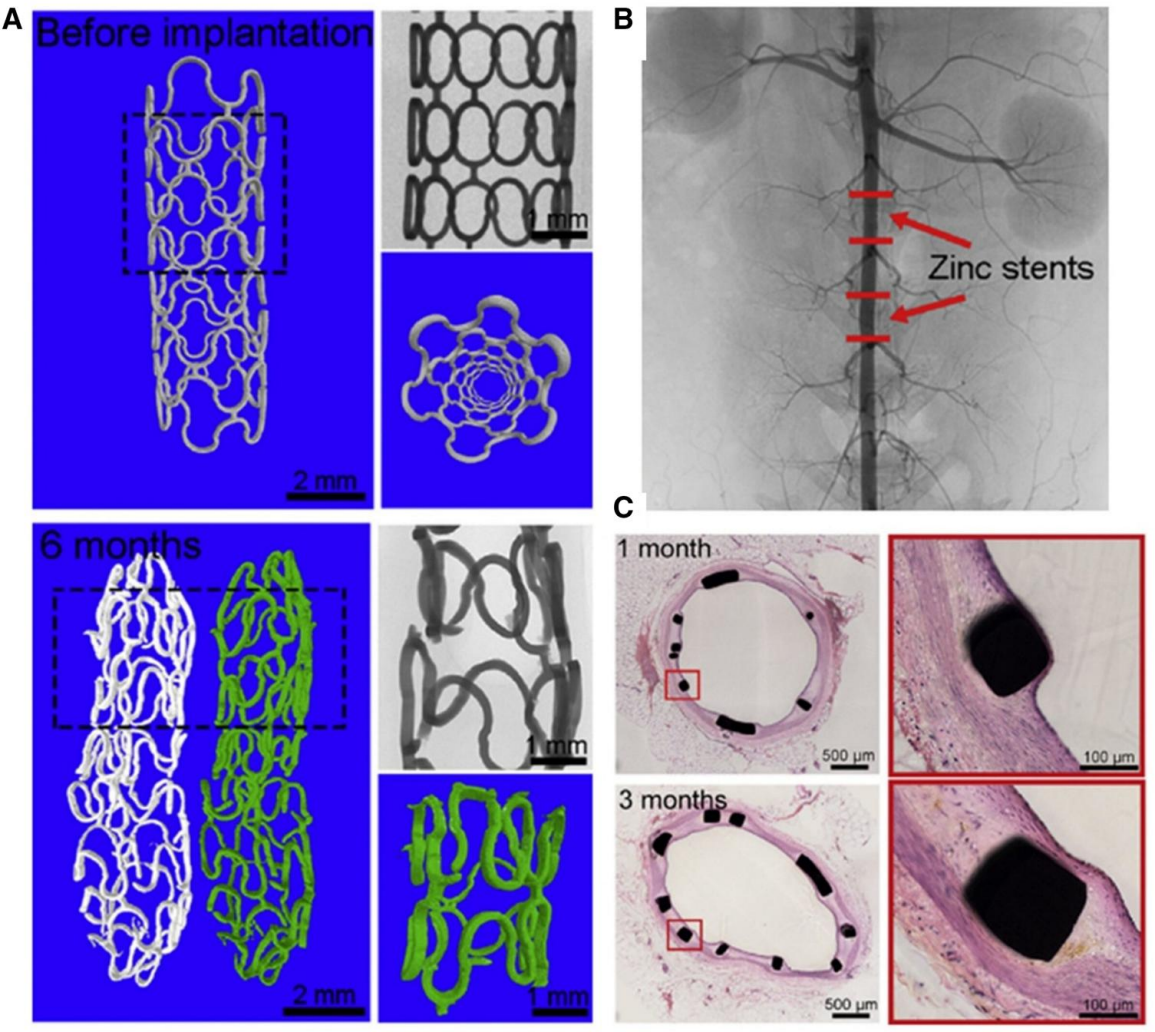
Preclinical testing of pure Zn stents. (**A**) Morphology, (**B**) implantation site and (**C**) histology of vascular response post-implantation [45].

Goldman et al. first explored Zn as a biodegradable cardiac scaffold material, implanting pure Zn filaments into the mouse aorta in 2013. Long-term implantation experiments confirmed that Zn could safely degrade in mice over extended periods and was suitable for stenting applications [44]. In 2017, Zheng et al. first implanted pure Zn stents (with a wall thickness of 165 μm) into the abdominal aorta of rabbits. They evaluated the biological response from 3 days to one year after implantation (Figure 4) and demonstrated that Zn exhibited a suitable degradation rate and good biocompatibility in the rabbit aorta [45]. In 2019, Zhang et al. implanted biodegradable Zn-0.8Cu stents in porcine coronary arteries. Their study monitored biological responses such as endothelialization, degradation, and inflammatory reactions for up to 24 months, confirming that Zn-0.8Cu alloys had suitable degradation rates, had structural integrity for six months, and caused minimal inflammation or thrombosis (Figure 5) [46].

However, the mechanical properties of pure Zn are insufficient to provide the necessary support as a scaffold for atherosclerosis. To address this issue, researchers have improved the mechanical properties of Zn-based stents through alloying. Fundamentally, the degradation behavior, localized toxicity, and mechanical properties of Zn-based stents are intricately linked to their microstructural features, such as grain size, crystallographic texture, and the distribution of secondary phases [47]. Recent advancements over the past two years have demonstrated that these critical microstructures can be precisely tailored through novel alloying strategies combined with advanced preparation methods [48]. For instance, sophisticated thermo-mechanical processing, severe plastic deformation, and other innovative techniques have been successfully employed to fabricate high-strength Zn alloys with ultrafine-grained structures [49]. These new preparation methods not only significantly enhance the ultimate tensile strength and ductility—thereby meeting the rigorous radial support demands of vascular scaffolds—but also promote a more uniform degradation profile, which helps to avoid sudden bursts of localized Zn^2+^ release [50]. Consequently, synergizing novel alloying elements with advanced manufacturing processes to optimize microstructural design serves as a crucial pathway to simultaneously address the mechanical shortcomings and mitigate the potential neurotoxicity of Zn-based stents. These efforts have led to the development and successful animal implantation of multiple degradable Zn alloy stents, including Zn-0.02Cu (wt%), Zn-0.8Cu (wt%, with a scaffold wall thickness of 100 μm), and Zn-3Ag (wt%) [51]. Overall, these *in vivo* studies revealed the potential for mild intimal hyperplasia and inflammatory reactions, but not thrombosis, confirming good biocompatibility. They also demonstrated these stents maintained an appropriate degradation rate, laying the groundwork for future clinical studies of degradable Zn alloy stents. Still, more research is needed to better understand the effects of Zn alloy material design on *in vivo* corrosion and biocompatibility.

While the development biodegradable Zn alloys has progressed rapidly—from the initial studies where Zn wires were implanted into the mouse aorta to experiments involving metal bare stents implanted in animal models—there have yet to be clinical trials of biodegradable cardiovascular stents made of Zn or Zn alloys [52].

### Considerations for intracranial application of Zn-based stents

Zn plays a vital role in the brain, participating in a range of biological processes that include neuronal growth, neurogenesis, and the regulation of synaptic function. As an essential cofactor, Zn facilitates numerous enzymatic reactions within the nervous system, from neurotransmitter production to intracellular signaling pathways. Regulating zinc concentration in the nervous system is necessary for preserving brain function, as either a surplus or deficiency in zinc levels can provoke neurological disturbances. Excessive accumulation of Zn can lead to localized neuronal toxicity, whereas insufficient availability of Zn can impair cognitive function and neural health [53].

The beneficial attributes of zinc supplementation in neuroinflammatory disease settings highlight its potential applicability in specialized medical devices, such as biodegradable intracranial stents, for cerebral vascular interventions [54]. In stroke, supplementation of Zn appears to play a role in mitigating damage; potentially contributing to BBB stabilization and, in doing so, reducing risks associated with inflammation and cerebral edema. Nevertheless, while administering Zn orally or systemically is beneficial for neurotransmitter activity, neuronal development, and BBB integrity, utilizing Zn alloys for stents in cerebral applications introduces unique challenges. Primarily, the degradation rate of Zn-based stents must be carefully managed to avoid rapid release of zinc ions, which could disrupt the surrounding neural environment. Rapid release of Zn may lead to localized neurotoxicity, incite inflammatory responses, and impair regular brain tissue functions, particularly if the BBB is compromised since BBB transport is the primary mechanism for regulating levels of Zn in the brain. Therefore, to facilitate the safe application of Zn alloy stents in brain vessels, refining the material design and alloy composition is needed to restrict ion release to within a tolerable threshold. Additional interventions such as surface coating may be necessary to further regulate zinc ion release, ensuring the stents maintain stability and compatibility within the neural environment over extended periods [55, 56].

**Figure 6:**
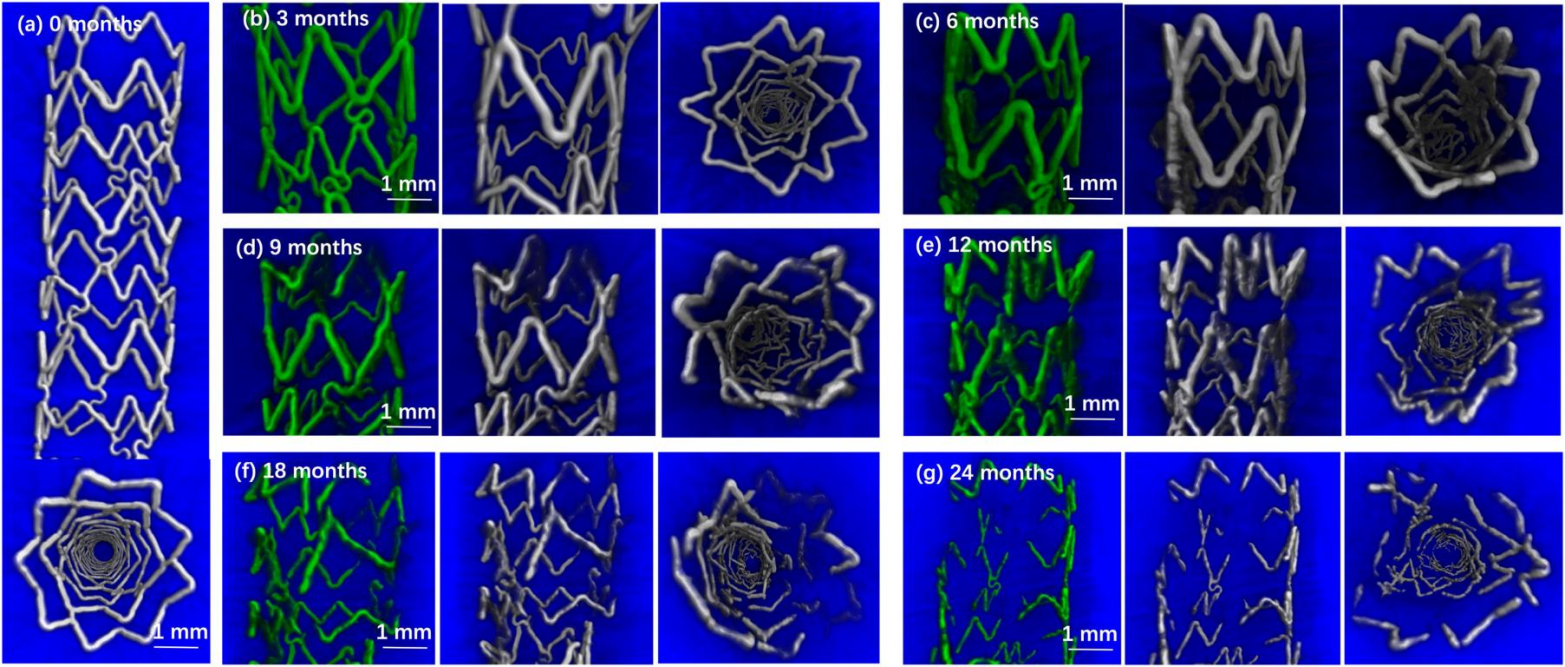
Time-resolved 3D micro-CT visualization of Zn-Cu stent degradation. (**A**) 3D micro-CT image of the Zn-Cu stent before implantation, and selected 3D images after (**B**) 3, (**C**) 6, (**D**) 9, (**E**) 12, (**F**) 18 and (**G**) 24 months post-implantation. The green images show degradation products at each time-point (front view) and the white images shows residual zinc with degradation products (both front and top views) [46].

Cerebral arteries, with their intricate curvature, require stents that can adapt to their shape without hindering blood flow or risking vessel injury, requires stents to exhibit both high flexibility and structural resilience [57–59]. Moreover, while research into Zn alloys has revealed favorable degradation and biocompatibility properties for coronary stents, further development is required to optimize these materials for cerebral use. Specifically, further control of the degradation rate of Zn stents is needed to prevent excessive ion release that could interfere with the brain’s function and cause neuroinflammation or cytotoxicity [60]. Zn-alloy stent should provide mechanical support for 3–6 months and fully degrade within 12–18 months to prevent vascular instability or prolonged inflammation caused by excessively fast or slow degradation. The physiological concentration of Zn^2+^ in CSF is approximately 150–300 nM, and local concentrations exceeding 1–5 µM may induce neurotoxicity. Therefore, alloy optimization is required to regulate degradation rates, and surface coatings should be applied to control ion release. Future work should aim to refine Zn alloy formulations and apply surface treatment techniques to achieve more desirable degradation properties, thereby ensuring safe and effective implementation of Zn stents in the cerebral vasculature (Figure 6).

## Degradable Mg-based alloy stents

### Mg-based alloys and Mg in the human body

Degradable Mg alloys possess excellent biocompatibility and strong mechanical support, effectively minimizing long-term complications such as intimal hyperplasia, restenosis, and late thrombosis. Mg is an essential major element in the human body, with approximately 25 g normally present. The concentration of Mg^2+^ in plasma ranges from about 0.70–1.05 mmol/L, and the recommended daily intake is around 350 mg [61]. Mg has multiple physiological functions that are closely linked to longevity and overall health. It activates numerous enzymes in the body, regulates nerve excitability, helps to maintain the stability of nucleic acid structures, and participates in protein synthesis, muscle contraction, and thermoregulation [62].

Mg exhibits diverse effects on cardiovascular function. It can lower blood pressure, treat acute myocardial infarction, and prevent atherosclerosis, making Mg-based alloys an ideal material for cardiovascular stents. Mg^2+^ inhibits the activity of Ca^2+^ influx and efflux in the sarcoplasm through antagonism, thereby preventing cardiac arrhythmias that can result from mitochondrial Ca^2+^ overload in cardiomyocytes. Additionally, Mg reduces systemic and pulmonary vascular resistance, decreases catecholamine release, and consequently, lowers blood pressure [63]. An increase in extracellular Mg decreases arterial tension and enhances the effects of vasodilatory substances. Mg can also improve local myocardial blood flow by alleviating coronary artery spasms. Furthermore, Mg affects blood coagulation, inhibits platelet aggregation, decreases high-density lipoprotein (HDL) levels, and prevents lipid deposition on arterial walls, thereby lowering the risk for atherosclerosis [64, 65].

However, the degradation rate of Mg-based alloys significantly limits their application in biodegradable stents. In bodily fluids containing chloride ions (Cl^−^), Mg-based alloys degrade too quickly, leading to the accumulation of degradation products in local tissues, which can cause intimal hyperplasia. Surface oxides of Mg-alloy stents gradually dissolve to Mg^2+^ ions in the body fluid environment and are degraded to oxide particles by phagocytosis and enzymatic degradation. Furthermore, rapid degradation can result in the scaffold losing its mechanical integrity and radial support prematurely, ultimately resulting in scaffold failure and vessel retraction [66]. To address these critical challenges, recent surface modification strategies have evolved from simple physical barriers to functionalized bioactive coatings. For instance, incorporating copper into biodegradable magnesium alloy stents via a Cu (II)-eluting coating has been shown to synergistically enhance prolonged durability and promote rapid re-endothelialization, thereby significantly improving the long-term vascular compatibility of the implants [67]. In addition, combination strategies are emerging as a powerful tool for alloy design and surface functionalization. A recent study demonstrated that combining Ta ion implantation with a functionalized polymer coating on vascular stents not only effectively regulates the degradation kinetics but also imparts multifunctional properties, offering a comprehensive solution to the biocompatibility and mechanical challenges of biodegradable metals [68].

### Early clinical application of degradable Mg-based alloy bare stents

Early studies of degradable Mg-based alloy bare stents demonstrated good biocompatibility and therapeutic efficacy. However, these studies also revealed several challenges, such as a degradation cycle too short to align with the effective treatment period and a high rate of restenosis in the late stage after stent implantation. These issues provided the basis for further development to improve Mg alloy stents [69].

**Figure 7:**
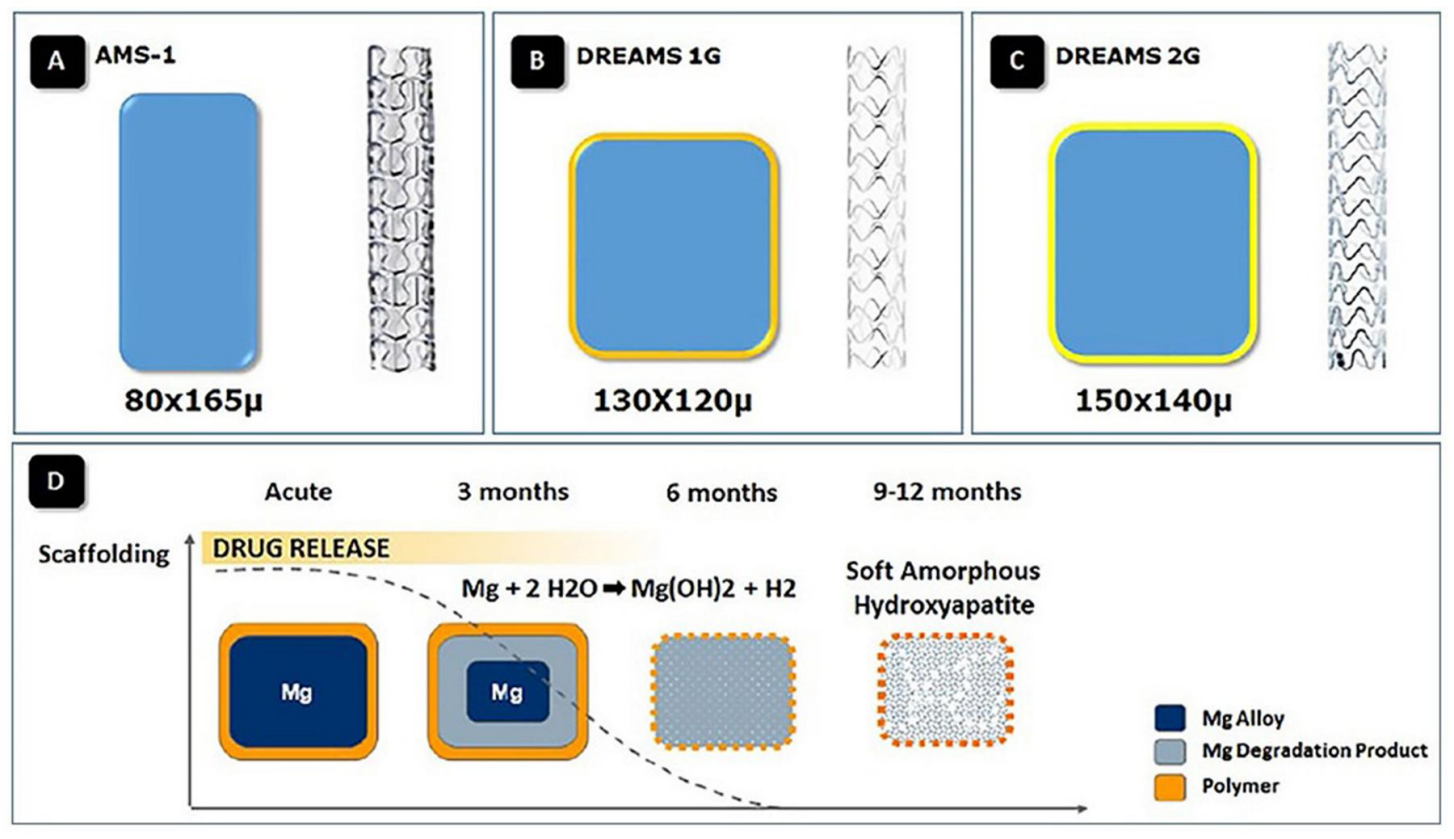
Technological advancements of four generations of magnesium alloy stents from Biotronik: (**A**) AMS, (**B**) DREAMS 1G, (**C**) DREAMS 2G and (**D**) drug-eluting stent drug release kinetics and degradation process [73].

In 2003, Heublein et al. [70] first used magnesium alloy AE21 (a Mg-Al-rare earth alloy) to fabricate coronary stents, which were implanted into the coronary arteries of pigs. The results showed that while the AE21 stents led to neointimal hyperplasia in the early stages post-implantation, there was minimal occurrence of thrombosis and inflammation [71]. In 2005, Mg alloy stents entered clinical use for the first time [72], specifically for use in peripheral arteries. Campos et al. used the Mg-alloy stent AMS (Biotronik, Berlin, Germany) to treat 20 patients with critical lower limb ischemia (Figure 7A) [73]. The results revealed a vascular patency rate of 89.5% at 3 months. However, subsequent research revealed that the 6-month patency rate of Mg alloy stents was significantly lower than that of conventional angioplasty (31.8% versus 58.0%), which was attributed to premature stent degradation, which can lead to insufficient support and suboptimal long-term outcomes. Erbel et al. [58] applied degradable Mg alloy stents to treat coronary artery occlusions, enrolling 63 patients and implanting a total of 71 degradable Mg stents. Patients were followed up with intravascular ultrasound (IVUS) and angiographic techniques. The results showed that the long-term patency rate of the Mg stents was lower than anticipated. Although degradable Mg stents achieved comparable short-term outcomes to traditional inert metal stents, their long-term efficacy remained unsatisfactory [58].

### Commercialization of degradable Mg-based stent products

Inspired by the clinical trial results of degradable Mg alloy bare stents, researchers extended the stent’s degradation period and incorporated drug coatings to improve outcomes. German company Biotronik altered the design of the degradable Mg alloy bare stent (AMS) and added an antiproliferative drug, paclitaxel, to the stent surface, a stent commercialized as DREAMS 1G (Figure 7) [73]. Compared to the bare stent, DREAMS 1G offers improved mechanical properties and a slower degradation rate, providing extended mechanical support. Furthermore, the eluted paclitaxel effectively inhibits endothelial proliferation.

In 2016, Haude et al. [62] reported the first clinical study of this stent enrolling 46 patients. Their findings revealed that the DREAMS 1G stent exhibited less late lumen loss compared to the bare stent, with a target lesion revascularization (TLR) rate of 4% (2/46) at 6 months. The stent essentially completed its degradation within 6 months without serious adverse events, e.g. cardiac death or stent thrombosis. These results also reveal the significant potential of applying drug elution to degradable magnesium stent design [74, 75]. Biotronik then developed a second-generation drug-coated degradable magnesium alloy stent, DREAMS 2G, which incorporates rapamycin, a more potent antiproliferative agent, on the stent surface.

In 2016, the Magmaris^®^ biodegradable Mg alloy stent received CE marking, becoming the first clinically approved magnesium stent. The stent is designed to elute sirolimus and degrade circa 12 months. The latest clinical data shows that the target lesion failure rate of the Magmaris^®^ degradable Mg-based stent is 4.7%, indicating a favorable safety profile [76].

**Figure 8:**
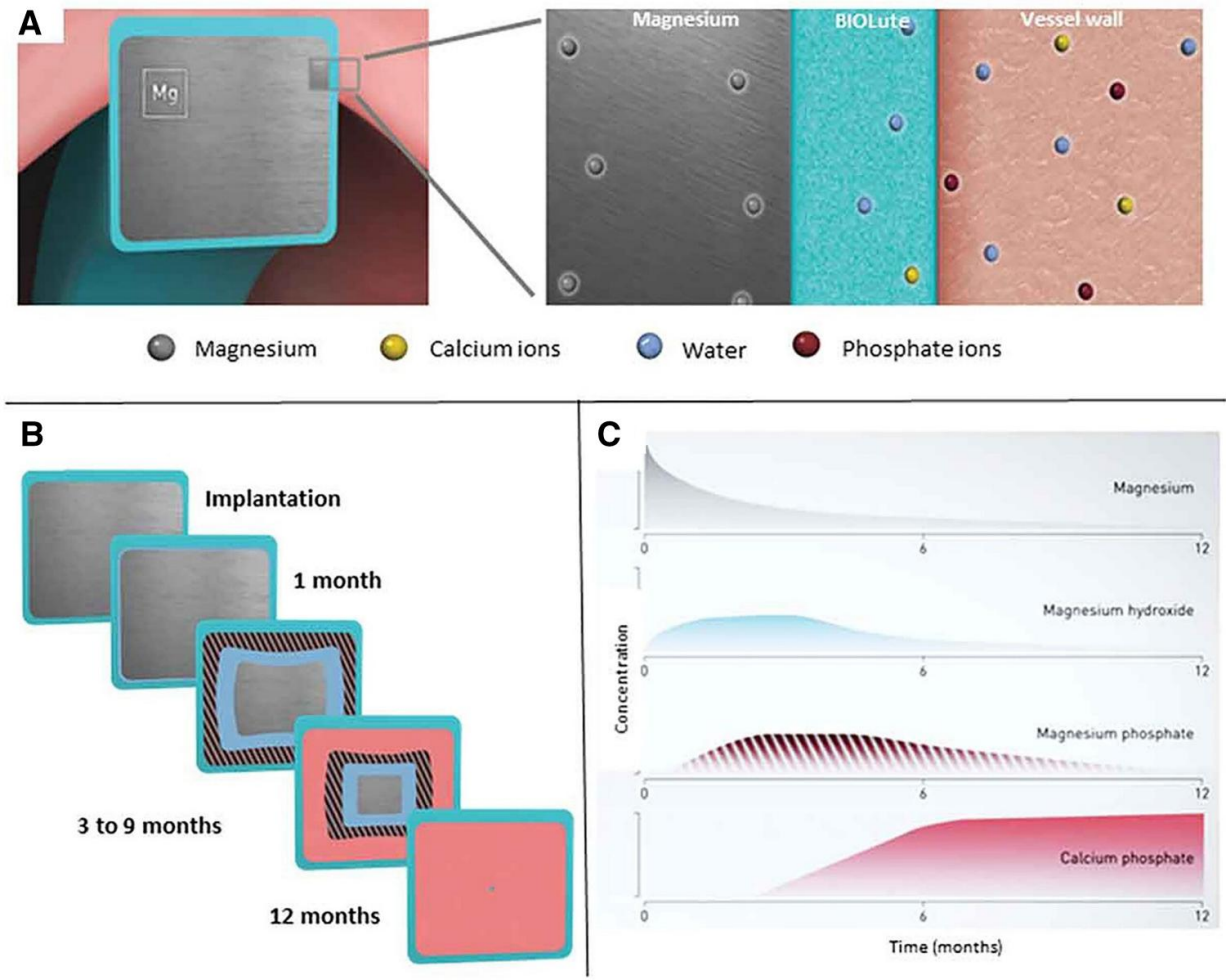
Magmaris resorption process. (**A**) Water and ions, e.g. calcium and phosphate, penetrate the coating to create a hydrated layer near the magnesium backbone. (**B**) Magnesium resorption begins at the surface and progresses inward, leaving an amorphous calcium phosphate residue. (**C**) Magnesium is resorbed in the form of magnesium hydroxide and magnesium phosphate [65].

Recent clinical advancements have further solidified the reliability and efficacy of magnesium-based stents. Notably, the BIOMAG-I study evaluated the third-generation sirolimus-eluting resorbable magnesium scaffold, DREAMS 3G. The 12-month follow-up results demonstrated an exceptionally low late lumen loss of 0.24 ± 0.36 mm after complete scaffold resorption, accompanied by a 0% incidence of definite or probable scaffold thrombosis [77]. These compelling data highlight the improved radial force and prolonged scaffolding capacity of DREAMS 3G, confirming its reliability as a highly competitive alternative to permanent DES. A comprehensive comparison between the outstanding 12-month outcomes of the DREAMS 3G magnesium scaffold and the upcoming long-term, large-cohort data of the IBS stent will provide critical clinical benchmarks, further guiding the material selection and clinical translation of biodegradable metal stents for diverse vascular applications (Figure 8).

### Considerations for intracranial applications of Mg-based stents

In evaluating Mg stents for use within the brain, it is essential to focus on the distinctive features and structural demands of cerebral vessels. Unlike cardiovascular vessels, cerebral arteries are uniquely characterized by greater curvature and significantly narrower diameters, with some measuring just a few millimeters across. Furthermore, cerebral arteries lack the external elastic membrane and supportive tissues that surround larger vessels, which coupled with their thinner walls, make them particularly vulnerable to stress under blood flow pressure [78]. These structural traits of cerebral arteries indicate that a stent designed for intracranial application must be highly adaptable to the vessel’s shape, offering enough flexibility to follow its complex curves without imposing undue mechanical stress. Adjusting degradation rate and customizing support strength are both critical for addressing the needs for flexibility and transience in ischemic cerebrovascular treatments within an environment as sensitive as the brain [79].

Mg alloys have a fast degradation rate and their degradation products (Mg^2+^ and H_2_) are considered to have good biocompatibility. Mg-alloy stents have been significantly improved in terms of corrosion resistance and degradation controllability by alloy optimization (e.g. Mg-Zn-Ca, Mg-Nd-Zr) and surface modification (e.g. PLGA, PLLA, MgO coating). In the coronary field, the DREAMS 2G Mg-alloy stent has entered the BIOSOLVE-II clinical trial. For intracranial applications, the hydrogen released during the degradation of magnesium can rapidly diffuse into the surrounding tissue or expelled through the skin without the formation of a local air embolism. However, Mg alloys still need to be further optimized for degradation homogeneity and fatigue resistance to ensure their long-term safety in high shear stress intracranial vessels. Currently, Mg-based stents are still in the exploratory stage for intracranial applications, but their potentially good degradation properties and neurological safety, as well as extensive clinical research demonstrating the safety and efficacy of Mg-alloy stents in various cardiovascular contexts, support their potential adaptation to the more delicate structures of cerebral vessels. In this way, magnesium alloy stents represent a forward-looking approach for addressing the specific demands of intracranial treatment.

In the specific context of cerebrovascular interventions, the application of bioabsorbable elastomer-coated magnesium alloy devices—such as coils designed for treating saccular cerebrovascular aneurysms—highlights the potential of soft-material surface modifications. These elastomer coatings can effectively buffer the rapid degradation of the underlying magnesium substrate while ensuring high conformability and safety in delicate neurovascular environments [80].

### Functionalized stents

A recent study by Zhang et al. [81] revealed the potential for Mg stents to provide neuroprotection after acute ischemic stroke (AIS). This research investigated the hypothesis that a localized release of Mg^2+^/H_2_ from Mg metal into the bloodstream could offer synergistic neuroprotection against reperfusion injury in distal cerebral ischemic tissues. Zhang et al. implanted Mg wires in the common carotid artery of rats after transient middle cerebral artery occlusion (MCAO). Notably, Mg wire implantation improved neurological behavior, decreased neuronal damage, stabilized the blood-brain barrier and enhanced cerebral blood flow. Moreover, these improvements corresponded with an increase in Mg and H_2_ concentration in both the blood and brain, which further supported this mechanism of neuroprotection *in vivo*. The significance of their work is that it is the first proof-of-concept study on biodegradable neuroprotective stents, which may shed light on future designs of novel, multi-functional cerebrovascular stents. This work also used and proposed a novel *in vivo* model for evaluating neuro-compatibility. They combined transient MCAO with a wire insertion to conduct pioneering investigations of *in vivo* neuroactivity of biodegradable Mg metals. Building upon this concept, recent advancements have successfully translated Mg-mediated neuroprotection into actual stent designs. For instance, a novel 3D-printed poly(L-lactide-co-ε-caprolactone) (PLCL) composite stent incorporated with MgSO4 particles was developed to achieve a sequential release of Mg^2+^ ions. This staged release profile was specifically designed to provide neuroprotection that aligns with the treatment window for AIS. *In vivo* evaluations demonstrated that the eluted Mg^2+^ effectively antagonized Ca^2+^ overload and scavenged reactive oxygen species (ROS), which significantly reduced the infarct volume and maintained BBB integrity. This progress represents a significant leap from proof-of-concept wires to functionally robust neuroprotective stents, further confirming the immense therapeutic potential of Mg-based interventions in cerebrovascular applications [82].

Considering the potential neurotoxicity associated with the degradation products of Fe and Zn, Mg currently emerges as the most promising candidate for neurovascular applications. To leverage this unique advantage, future strategies could consider incorporating magnesium ions as functionalized coatings on Fe- and Zn-based alloy stents, which would not only mitigate their localized toxicity but also provide synergistic neuroprotection to the neurovascular unit (NVU) [69].

## Effects of degradation by-products on the neurovascular unit

Following the implantation of biodegradable metal stents (Mg-, Zn- and Fe-based) in cerebrovascular applications, the degradation-released ions and microenvironmental changes exert profound biological effects on the NVU.

The Mg^2+^released from Mg-based stents acts as a natural calcium antagonist, which can inhibit NMDA receptor-mediated calcium overload and reduce neuronal damage [81]. A recent study by Zhang et al. [82] in 2025 demonstrated that a 3D-printed Mg-based stent, through the sequential release of Mg2+, effectively enhanced the expression of tight junction proteins in endothelial cells (ECs) and stabilized BBB. Concurrently, the byproduct H2, functioning as a selective antioxidant, scavenges ROS and acts synergistically with Mg^2+^ to improve local cerebral blood flow [81]. Zn2+ exhibit a biphasic regulatory effect on ECs. Recent advancements in 2025 revealed that Zn^2+^ suppresses NF- κB activity via the IKB signaling pathway, thereby driving the polarization of microglia from a pro-inflammatory M1 phenotype toward a reparative M2 phenotype [83]. Furthermore, Zn^2+^ can activate the STAT3-FOXO3a-SOD2 axis to restore microglial mitochondrial metabolic homeostasis, indirectly promoting neuronal survival [84]. Fe^2+^ generated from the degradation of Fe-based stents can activate autophagy in smooth muscle cells (SMCs) via the AMPK/mTOR pathway, which inhibits excessive proliferation and prevents in-stent restenosis [85]. However, an excess of Fe^2+^ is prone to inducing neuronal lipid peroxidation and ferroptosis via the Fenton reaction [86, 87].

The alkaline environment generated during magnesium degradation can neutralize acidosis in ischemic tissues. This buffering effect assists neurons in maintaining the ‘depolarization-induced alkalization’ mechanism, ensuring their electrophysiological activity [88].

## Conclusion and outlook

Degradable Fe-based metal stents exhibit excellent mechanical properties, but their degradation rate is too slow for this application. Through surface modification, researchers have successfully regulated the degradation rate, and these stents have now entered the clinical trial stage. Mg-based stents, which are known for their outstanding biocompatibility and important physiological roles in the cardiovascular system, instead suffer from rapid degradation that results in insufficient mechanical properties. However, after extensive development, degradable Mg-based stents now have substantial clinical promise. Meanwhile, degradable Zn-based stents, an emerging research focus, have promising mechanical properties and a suitable corrosion rate, but they are still in the preclinical stage of development. Overall, studies to date suggest degradable metal stents are a promising interventional treatment for cardiovascular and cerebrovascular diseases. With regards to cardiovascular stents, the clinical data reported thus far consist of small-scale studies and simple vascular lesion implants. Large-scale clinical trials are still needed for validation. With regards to cerebrovascular applications, Mg-, Fe- and Zn-based stents would benefit from further optimization of degradation rate and biocompatibility through alloying design and surface modification. A detailed comparison contrasting the specific cardiovascular and cerebrovascular requirements for these degradable metals is summarized in Table 1.

**Table 1:** Comparison of requirements for Fe-, Zn-, and Mg-based stents in cardiovascular versus cerebrovascular.

Target	Clinical needs	Fe-based stents	Zn-based stents	Mg-based stents	Reference
Cardiovascular	Needs sustained mechanical support	High radial support (ultrathin struts)	Mechanical enhancement via alloying	Prolonged radial support against rapid degradation	[1, 20, 21, 10, 41, 45, 58–60]
Cerebrovascular	Needs high flexibility and strict neuro-compatibility	High flexibility and MRI compatibility;	Strict ion threshold (<1-5 µM); Precise controlled-release coatings	Scavenge ROS & preserve BBB; Safe H₂ diffusion	[28, 35, 36, 54, 73, 58, 76]

To provide a more quantitative perspective on material selection, Table 2 summarizes the mechanical properties and degradation rates of representative mainstream degradable metals, explicitly highlighting their current status against the mechanical benchmarks required for cerebrovascular stents.

**Table 2:** Mechanical and degradation properties of biodegradable metals for cerebrovascular stents.

Target	Representative	UTS (MPa)	YS (MPa)	Modulus (GPa)	Elongation (%)	Degradation rate (mm/year)	References
Cerebrovascular benchmark	—	> 300	∼200	∼ 200	15–18	Matches healing window	[10]
Fe-based	Nitrided Fe	> 600	> 300	∼ 200	10–20	0.1–0.5	[25]
Zn-based	Zn-Mg-Mn/Zn-Cu-Ag	300–414	200–300	80–100	20–30	0.1–0.5	[47, 48]
Mg-based	WE43/Composites	250–300	150–200	40–45	10–17	1.0–3.0	[89, 90]

This direction will continue to be a major focus for future research on degradable metallic cerebrovascular stents.

A critical challenge in the clinical translation of biodegradable metal stents remains the discrepancy between *in vitro* evaluations and *in vivo* performance. Deriving a universal mathematical ‘conversion factor’ between the two is highly complex due to the dynamic physiological environment. According to comprehensive comparative reviews [91], *in vitro* degradation rates tested in simple SBF frequently overestimate true *in vivo* degradation—sometimes by a factor of 2–4 for Mg-based alloys. This discrepancy primarily arises because *in vivo* protein adsorption and subsequent fibrous encapsulation act as protective barriers, significantly restricting ion exchange and fluid penetration at the stent interface. Conversely, the high shear stress characteristic of the cerebrovascular environment may mechanically accelerate corrosion, complicating this protective effect. Currently, studies indicate that modifying *in vitro* testing setups by incorporating protein-supplemented media under dynamic flow and precise CO2 buffering can narrow the *in vitro*-to-*in vivo* degradation ratio closer to 1:1 [92]. Establishing such standardized, hemodynamically accurate dynamic models is an absolute prerequisite for predicting the reliable *in vivo* lifespan of cerebrovascular stents. These challenges are summarized in Table 3.

**Table 3:** Critical unresolved issues prior to clinical translation of biodegradable cerebrovascular stents

Critical issues	Current challenges	Future directions
In *vitro* vs. in *vivo*	Static assays in simple SBF overestimate degradation and fail to mimic high-shear cerebrovascular hemodynamics.	Establish standardized, hemodynamically accurate dynamic models incorporating protein-supplemented media and precise CO₂ buffering.
NVU toxicity	The long-term toxicological mechanisms of degradation by-products on the delicate NVU remain unclear.	Systematically quantify local neurotoxicity and long-term NVU compatibility.
Neuroprotection	Therapeutic effects are not precisely aligned with the narrow ischemic stroke treatment window.	Develop intelligent, staged-release composite systems for targeted neuroprotection.
Architecture and imaging	Cerebral vessels require extreme conformability, while severe MRI artifacts hinder non-invasive monitoring.	Optimize highly flexible, MRI-compatible architectures and advanced elastomeric coatings to control degradation kinetics.

In conclusion, translating biodegradable metal stents to intracranial applications still faces critical research gaps. Primarily, the unpredictable interference of complex, high-shear cerebrovascular hemodynamics on *in vivo* degradation profiles remains unresolved [93]. Furthermore, the long-term toxicological mechanisms of degradation by-products on the delicate NVU require systematic quantification [33]. To bridge these gaps, future priority directions must shift toward developing intelligent, staged-release composite systems capable of delivering targeted neuroprotection precisely aligned with the ischemic stroke treatment window [82]. Additionally, optimizing highly flexible, MRI-compatible architectures and designing advanced elastomeric coatings to strictly control degradation kinetics will be paramount for ensuring conformability and safety in the cerebral vasculature.

## Data Availability

The data that support the findings of this study are available from the corresponding author upon reasonable request.
